# Severe Glutathione Deficiency, Oxidative Stress and Oxidant Damage in Adults Hospitalized with COVID-19: Implications for GlyNAC (Glycine and *N*-Acetylcysteine) Supplementation

**DOI:** 10.3390/antiox11010050

**Published:** 2021-12-27

**Authors:** Premranjan Kumar, Ob Osahon, David B. Vides, Nicola Hanania, Charles G. Minard, Rajagopal V. Sekhar

**Affiliations:** 1 Translational Metabolism Unit, Section of Endocrinology, Diabetes and Metabolism, Department of Medicine, Baylor College of Medicine, Houston, TX 77030, USA; premranjan.kumar@bcm.edu (P.K.); ob.osahon@bcm.edu (O.O.); david.vides@bcm.edu (D.B.V.); 2 Section of Pulmonology, Critical Care and Sleep Medicine, Department of Medicine, Baylor College of Medicine, Houston, TX 77030, USA; hanania@bcm.edu; 3 Institute of Clinical and Translational Research, Department of Medicine, Baylor College of Medicine, Houston, TX 77030, USA; minard@bcm.edu

**Keywords:** COVID-19, glutathione, oxidative stress, oxidant damage, GlyNAC

## Abstract

Humanity is battling a respiratory pandemic pneumonia named COVID-19 which has resulted in millions of hospitalizations and deaths. COVID-19 exacerbations occur in waves that continually challenge healthcare systems globally. Therefore, there is an urgent need to understand all mechanisms by which COVID-19 results in health deterioration to facilitate the development of protective strategies. Oxidative stress (OxS) is a harmful condition caused by excess reactive-oxygen species (ROS) and is normally neutralized by antioxidants among which Glutathione (GSH) is the most abundant. GSH deficiency results in amplified OxS due to compromised antioxidant defenses. Because little is known about GSH or OxS in COVID-19 infection, we measured GSH, TBARS (a marker of OxS) and F2-isoprostane (marker of oxidant damage) concentrations in 60 adult patients hospitalized with COVID-19. Compared to uninfected controls, COVID-19 patients of all age groups had severe GSH deficiency, increased OxS and elevated oxidant damage which worsened with advancing age. These defects were also present in younger age groups, where they do not normally occur. Because GlyNAC (combination of glycine and *N*-acetylcysteine) supplementation has been shown in clinical trials to rapidly improve GSH deficiency, OxS and oxidant damage, GlyNAC supplementation has implications for combating these defects in COVID-19 infected patients and warrants urgent investigation.

## 1. Introduction

Since 2019, the world has been in the grip of a pandemic caused by the novel severe acute respiratory syndrome coronavirus 2 (SARS-CoV-2) which causes an inflammatory viral pneumonia called coronavirus disease 2019 (COVID-19) [1,2]. Patients infected with COVID-19 can develop fever and respiratory symptoms and are often admitted to the hospital due to progressive dyspnea and systemic complications necessitating support measures ranging from supplemental oxygen to the need for mechanical ventilation and intensive care [3,4]. The COVID-19 pandemic is associated with episodic global surges (‘waves’) associated with large numbers of patients seeking hospitalization which places huge strains on healthcare staff, overruns hospitals and severely challenges healthcare systems as was witnessed globally with the recent delta variant. The discovery and rollout of COVID-19 vaccines were expected to boost herd immunity to rein in the raging pandemic, but viral mutations, vaccine hesitancy and vaccine non-availability have led to the rise of new strains of SARS-Cov-2 sequentially named β, δ, κ, μ, and the recent South African Omicron strain which has led to much global fear and concern due to its rapid international spread and has been designated by the World Health Organization as a variant of concern [5]. These newer strains appear to have variable vaccine resistance resulting in breakthrough infections even in vaccinated patients, and COVID-19 waves continue to surge even in heavily vaccinated countries as is currently occurring in the United Kingdom and the European Union. As a result, the global scientific and medical communities are urgently trying to prepare for and manage newer COVID-19 waves, as this cycle tends to repeat at periodic intervals. In a new development, a recent study reports new data from an analysis of 13638 patients (with and without COVID-19) which suggests that these patients have an increased risk of death in the following 12-months [6]. While older adults (OA) > 65 years of age have a higher risk of hospitalization and death due to acute COVID-19 infection [7], this new study reports that post-COVID mortality in adults < 65 year of age is higher than those > 65 year of age [6]. The underlying reasons for this increase in post-COVID mortality are currently unclear but unrelated to cardio-respiratory etiology and attributed to COVID-19 related biological and physiological stresses [6]. Because COVID-19 is a highly dynamic and unpredictable disease it is urgently necessary to identify and target all mechanistic defects which may be associated with poor health in patients with acute COVID-19, and also in the post-COVID aftermath.

Oxidative stress (OxS) is a harmful condition caused by excess accumulation of reactive oxygen species and is linked to lung disease [8,9], heart disease [10,11], neurological disorders [12], diabetic complications [13], liver [14] and kidney diseases [15], and to the biology of the aging process [16,17]. Under physiological conditions, OxS is neutralized by antioxidants among which glutathione (GSH) is the most abundant endogenous intracellular antioxidant [18,19,20]. Conditions with the highest risk of complications (including mortality) as a result of COVID-19 infection include older age, diabetes and immunocompromised status [21,22,23,24,25], and all three conditions have in common a high risk of elevated OxS and GSH deficiency [18,19,20]. We have studied GSH deficiency and OxS in older humans, immunocompromised HIV patients and diabetic patients and have reported that correcting these defects with GlyNAC (combination of GSH precursor amino acids glycine, and cysteine provided as N-acetylcysteine) significantly improves multiple additional defects and boosts health [26,27,28,29,30,31,32]. Although GSH deficiency is proposed as the most likely cause for serious manifestations and death in COVID-19 [33], little is known of GSH adequacy, OxS, or oxidant damage in adults hospitalized with COVID-19. Therefore, we measured intracellular GSH concentrations and plasma OxS in hospitalized COVID-19 patients and report our findings here.

## 2. Materials and Methods

### 2.1. Study Approval

All subjects gave their informed consent for inclusion before they participated in the study. The study was conducted in accordance with the Declaration of Helsinki, and the protocol was approved by the Institutional Review Boards (IRB) at Baylor College of Medicine, and Harris Health System in Houston, TX, USA.

### 2.2. Participants

A total of 60 participants (25 women, 35 men; age range 21–85 years), admitted with COVID-19 (based on a PCR diagnosis) admitted to Ben-Taub General Hospital in Houston, TX were recruited and results compared to 24 uninfected historical controls from our prior studies conducted before the COVID-19 pandemic (pre-2019).

### 2.3. Study Details

The study involved a single blood draw at one time, collected within the first 24-h of admission, after which patients concluded their participation in the study. Clinical lab work (plasma liver profile, BUN, Creatinine, glucose, blood counts) was obtained from the hospital records on admission.

### 2.4. Outcome Measures

#### 2.4.1. Glutathione Concentrations and Oxidative Stress

RBC (red-blood cell) glutathione concentrations were measured in duplicate using the liquid chromatography (Waters ACQUITY UPLC System). Briefly, after blood was centrifuged, and plasma removed, 500 μL of red-blood cells were mixed immediately with 500 μL of chilled, isotonic monobromobimane buffer solution. This was subjected to 3 freeze-thaw cycles with liquid nitrogen to lyse RBCs. After being vigorously vortex-mixed, the whole blood–MBB mixture was incubated in the dark for the development of the glutathione-MBB derivative. Proteins were then precipitated with ice-cold 20% perchloric acid, and the supernatant fluid was analyzed for glutathione using the UPLC. To determine GSSG concentrations, the reducing agent dithiothreitol was added to convert RBC-GSSG to glutathione, and the sample was processed as described above to give concentrations of total-GSH. The GSSG concentrations were obtained by subtracting the glutathione value of the total GSH from the reduced GSH. Plasma markers of OxS (as Thiobarbituric acid reducing substances, Cayman Chemical, Ann Arbor, MI, USA), and oxidant damage (as F2-isoprostanes, 8-Iso-Prostaglandin-F2a, Cell Biolabs Inc., San Diego, CA, USA) were measured using ELISA assays.

#### 2.4.2. Plasma Biochemistry

Liver profile, creatinine, BUN, glucose and blood counts were obtained from the hospital admission records.

### 2.5. Statistical Methods

Biomarker measures are summarized by means with standard deviations. Summary statistics are stratified by cohort (Controls vs. COVID-19). A multiple linear regression model estimated the mean (95% CI, confidence interval) response. A separate model is fit for each outcome measure. Models include fixed effects for cohort, age group, and sex as well as all two-way and three-way interaction terms. The models test 8 specific hypothesis tests per measure. *p*-values are adjusted for multiple hypothesis tests using the Bonferroni correction only within each model. Statistical significance is assessed at the two-sided 0.05 level. Model assumptions were assessed by residual analysis.

## 3. Results

### 3.1. Age

Controls were 51.7 ± 20.2 years of age, and hospitalized COVID-19 patients were 51.0 ± 14.6 years of age. Study participants included (a) Young Adults (21–40 y) with COVID-19 (YA-C, N = 21) and uninfected Young Adults who served as controls (YA, N = 8); (b) Middle-Aged adults (41–60 year) with COVID-19 (MA-C, N = 21) and uninfected Middle-Aged adults who served as controls (MA, N = 8); (c) Older Adults (>60 year) in the COVID-19 group (OA-C, N = 18) and uninfected Older Adults as the control group (OA, N = 8).

### 3.2. Plasma Biochemistry

Laboratory results are shown in Table 1.

### 3.3. GSH Adequacy

**Figure 1:**
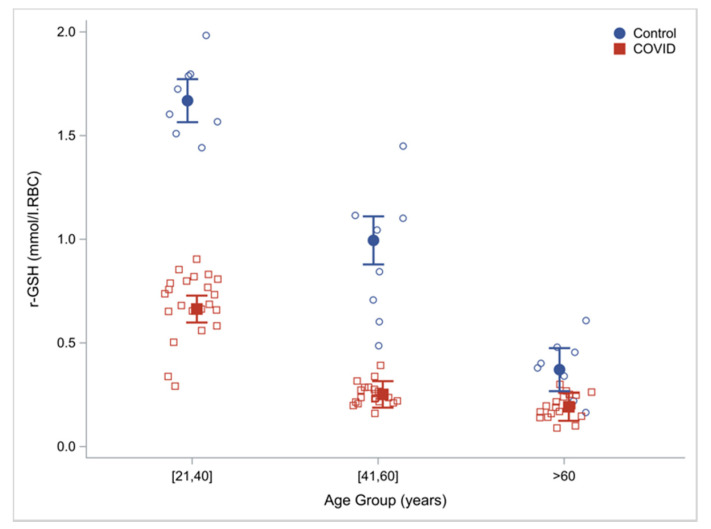
Intracellular reduced glutathione concentrations by age and cohort (COVID-19 patients vs. uninfected controls) with regression estimated means (95% CI) adjusting for cohort, age, group and sex.

Compared to controls, RBC concentrations of total-GSH (tGSH) and reduced-GSH (rGSH) in COVID-19 patients were 60% lower (Table 2). When these outcomes were analyzed in discrete age groups, compared to uninfected controls, COVID-19 patients had 60.2%, 74.7% and 48.3% lower RBC concentrations of reduced GSH in the young (21–40 years), middle-aged (41–60 years) and older humans (≥60 years) suggesting that GSH deficiency was present in young and middle-aged COVID-19 patients, and is more severe in older COVID-19 patients (Table 3). Within the COVID-19 group, there was an age effect with a progressive decrease in GSH concentrations with increasing age. (rGSH shown in Figure 1).

### 3.4. Oxidative Stress

#### 3.4.1. Oxidative Stress

**Figure 2:**
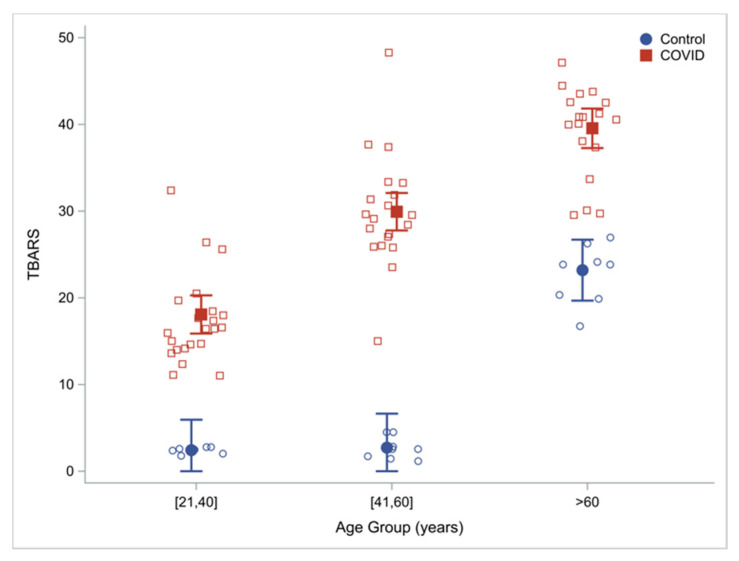
Oxidative stress: plasma TBARS by age group and cohort (COVID-19 patients vs. uninfected controls) with regression estimated means (95% CI) adjusting for cohort, age, group and sex.

Plasma concentrations of TBARS (lipid peroxidation) were measured as an index of OxS. Compared to controls, TBARS levels were 203% higher in COVID-19 patients (Table 2). When these outcomes were analyzed in discrete age groups, compared to uninfected controls, COVID-19 patients had 654%, 1007% and 70.6% higher concentrations of TBARS in the young (21–40 year), middle-aged (41–60 year) and older humans (≥60 year) respectively, suggesting that young and middle-aged COVID-19 patients have severe and significantly elevated OxS. Although uninfected older adults (OA) already have elevated OxS compared to younger adults [26,31], OxS in COVID-19 infected OA is higher than that in uninfected OA. Within the COVID-19 group, there was an age effect with a progressive increase in TBARS concentrations with increasing age (Table 3; Figure 2).

#### 3.4.2. Damage Due to OxS (Oxidant Damage)

**Figure 3:**
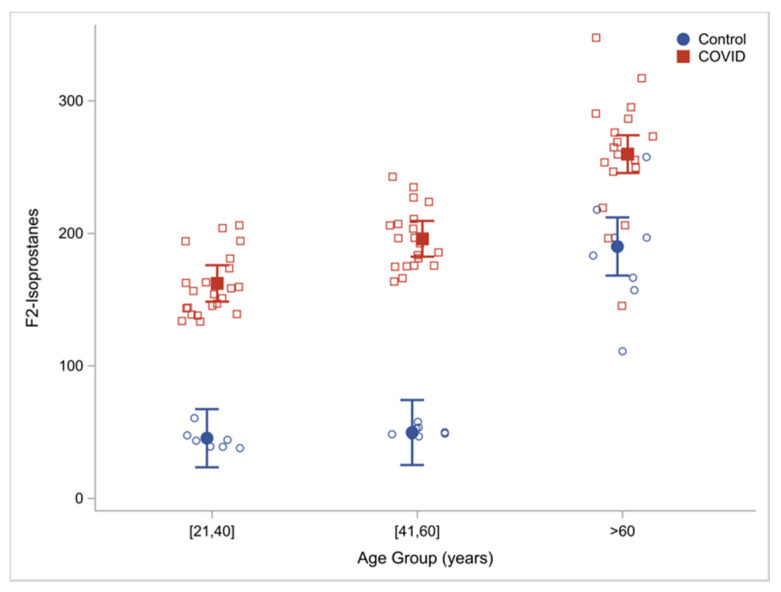
Damage due to oxidative stress: plasma F2-isoprostane concentrations by age and cohort (COVID-19 patients vs. uninfected controls) with regression estimated means (95% CI) adjusting for cohort, age, group and sex.

Oxidant damage was measured as plasma concentrations of F2-isoprostanes (F2-I). Compared to uninfected controls, plasma concentrations of F2-I were 115% higher in COVID-19 infected patients (Table 1). When these outcomes were analyzed in discrete age groups, compared to uninfected controls, COVID-19 infected patients had 257%, 294% and 37% higher concentrations of F2I in the young (21–40 year), middle-aged (41–60 year) and OA (≥60 year) respectively, suggesting that young and middle-aged COVID-19 patients had severely elevated markers of oxidant damage, and this was also more severe in OA with COVID-19 infection than uninfected OA. Within the COVID-19 group, there was an age effect with a progressive increase in F2-I concentrations with increasing age (Table 3; Figure 3).

## 4. Discussion

The key findings of this study are that (1) compared to uninfected controls, hospitalized COVID-19 patients have severe GSH deficiency, elevated OxS and increased oxidant damage; (2) these defects also occur in younger COVID-19 patients; (3) the magnitude of defects in COVID-19 patients increases with increasing age.

### 4.1. GSH Deficiency in COVID-19

Glutathione is the most abundant intracellular antioxidant tripeptide. Cellular synthesis of GSH occurs from 3 amino acids cysteine, glycine and glutamic acid in two discrete steps in the cytosol. GSH is present in multiple cellular components including the mitochondria, nucleus and endoplasmic reticulum [34] where it plays important roles in cellular protection and multiple pathways. For example, we have shown that depleting GSH in young wild-type mice results in mitochondrial dysfunction and that correcting GSH deficiency in old wild-type mice lowers reverses mitochondrial impairment, lowers OxS and insulin resistance [29].

GSH levels have been reported to be inversely related to multimorbidity in older adults (OA) [35]. It is established that OA in the geriatric age group have an increased prevalence of GSH deficiency [18,19,20,26,29,30]. Therefore, the findings of this study are interesting because they show a much more widespread prevalence of GSH deficiency in all age groups of adult humans hospitalized with COVID-19, especially in younger humans. This is an important discovery because younger humans are not expected to have GSH deficiency, but we found that patients in the 21–40 and the 41–60 year age groups had severe GSH deficiency compared to uninfected age-matched controls. OA with acutely infected with COVID-19 have the highest rates of hospitalization and mortality [7]. In our study, we found that OA hospitalized with COVID-19 had the lowest GSH concentrations, with GSH levels lower than uninfected age-matched control OAs suggesting that when older humans are infected with COVID-19, their GSH levels decline even further. Collectively these data indicate that GSH deficiency is highly prevalent in patients admitted to hospitals due to COVID-19. In previous clinical trials in OA, diabetic patients and in HIV-infected patients, we found and reported that GSH deficiency occurs as a result of decreased intracellular synthesis (due to deficiency of GSH precursor amino acids glycine and cysteine) [26,27,28,29,30,31,32]. Although the reasons for GSH deficiency in hospitalized COVID-19 patients are unclear, it could well be the result of a combination of factors including decreased synthesis and increased GSH utilization. In our prior trials [26,27,28,29,30,31,32], we found that supplementing GlyNAC (combination of glycine and *N*-acetylcysteine, a cysteine donor) for a relatively short period of 14 days improves/corrects GSH deficiency in OA, HIV-patients and diabetic patients, and these data suggest the possibility that similar supplementation of GlyNAC in COVID-19 patients could improve GSH deficiency, and warrants investigation. The implications of GlyNAC supplementation in COVID-19 infection are discussed in a subsequent section below.

### 4.2. Oxidative Stress and Oxidant Damage in COVID-19

Oxidative stress (OxS) is a harmful condition associated with cellular toxicity and organ dysfunction due to oxidant induced damage. OxS contributes to dysfunction affecting the lungs, heart, brain, liver, muscle, pancreas, and to abnormalities, such as inflammation and vascular dysfunction which are commonly present in COVID-19 and other conditions including aging, diabetes, HIV, Alzheimer’s disease, cardiovascular disease and more [8,9,10,11,12,13,14,15]. Therefore, the findings of this study that COVID-19 is associated with excessively elevated OxS and evidence of oxidant damage are important, as they could contribute to COVID-19 related injury and mortality. Targeting OxS and oxidant damage effectively could be key in improving health and survival in COVID-19 infected patients.

OxS originates from the accumulation of excess reactive oxygen species which are formed in mitochondria during the process of energy generation. Cells usually depend on antioxidants for protection from OxS and oxidant damage, and GSH is the most abundant intracellular antioxidant [18,19,20]. Therefore, GSH deficiency can amplify the destructive potential of OxS due to compromised antioxidant defenses. In uninfected humans, OxS tends to occur mainly in older humans (>60 year of age) and not in younger age groups. Indeed, the ‘free radical theory of aging’ was proposed in 1956 to suggest that elevated OxS in older humans could be responsible for the aging process [16]. Therefore, the observation in this study that COVID-19 infected patients in the young (21–40 year) and middle-aged (41–60 year) groups have severely elevated OxS and oxidant damage is important, as they could help explain the health deterioration associated with COVID-19 resulting in hospitalization and death. This is especially relevant in light of the recent COVID-19 surge with the delta variant which disproportionately affected younger humans, and that the <65 year age group has a higher rate of mortality in the 12-months following COVID-19 infection [6]. Certain populations are associated with a higher prevalence of elevated OxS (such as OA, immunocompromised HIV-infected patients and diabetic patients) and COVID-19 infection in such patients could result in a catastrophic increase in OxS resulting in health deterioration and is a probable reason why these groups are exceptionally vulnerable to adverse outcomes related to acute COVID-19 infection. In clinical trials in these populations of OA, HIV-patients and diabetic patients, we studied and reported that supplementing GlyNAC provides powerful, biologically relevant cellular protection from the harmful and toxic effects of OxS, without the risk of reductive stress [26,27,28]. Therefore, it is likely that GlyNAC supplementation could play an important role in combating OxS and oxidant damage toward protecting cellular health in COVID-19 patients and is discussed next.

### 4.3. Potential Benefits of GlyNAC Supplementation in COVID-19

Could GlyNAC supplementation have health benefits for patients with COVID-19? Older age, immunocompromised status (such as HIV infection) and diabetes are among pre-existing conditions most vulnerable to the ravages of COVID-19. In published clinical trials in OA, HIV-infected patients and diabetic patients, we reported that GlyNAC supplementation for 2-weeks rapidly improves GSH deficiency, OxS, and damage caused by OxS [26,27,28], and longer durations of supplementation correct these defects [30,31]. A computational analysis of therapeutic targets and discovery of potential drugs against SARS-Cov-2 identified GSH as a key potential candidate [36]. GSH has been reported to inhibit replication of the influenza virus (which causes a viral respiratory pneumonia with a high annual mortality rate in OA) [37], and we speculate that if boosting GSH can inhibit replication of the SARS-Cov-2 virus which causes COVID-19, this could be a gamechanger in the global fight against the COVID-19 pandemic. A small case study reported that increasing GSH levels improved dyspnea in 2 patients infected with COVID-19 [38], and NAC supplementation is reported to have a beneficial impact in ventilated COVID-19 patients [39]. However, the use of antioxidant supplements in COVID-19 should be used with caution due to the risk of inducing reductive stress, a condition excess lowering of reactive oxygen species can cause harm. In multiple clinical trials, we have shown that GlyNAC successfully lowers OxS, without triggering reductive stress. Additional benefits of GlyNAC supplementation comes from its ability to provide the vitally important amino-acids glycine and cysteine. Glycine is a 1-carbon metabolite and a methyl-group donor which is necessary for DNA synthesis, cellular reactions, brain, cartilage and cellular health. Indeed, methyl-group deficiency has been proposed as a potential mechanism for COVID-related complications [40]. Cysteine (from NAC) is critically important for supporting mitochondrial energy metabolism and donates a sulfhydryl group which is necessary for multiple cellular reactions and biosynthesis of metabolites. By virtue of its ability to provide glycine and cysteine and GSH, GlyNAC is referred to as representing a ‘power of three’. The benefits of GlyNAC supplementation go well beyond correcting GSH deficiency and OxS as it also improves inflammation, mitochondrial dysfunction, endothelial vascular dysfunction, insulin resistance, genotoxicity, autophagy/mitophagy and muscle strength as reported in human clinical trials [31,32]. This is relevant because similar defects are also reported in patients with COVID-19 [41,42,43,44,45,46,47,48]. Overall, the combination of the findings of this study, and the observations on the potential benefits of GlyNAC supplementation in prior clinical trials suggest a potentially beneficial role for GlyNAC supplementation in COVID-19 infected patients and supports the need for research studies to evaluate the impact of GlyNAC supplementation in COVID-19.

### 4.4. Study Limitations

The limitation of this study is that blood was collected one single time within 24 h of admission, and results should be interpreted with that caveat. Future studies are needed to determine whether GSH deficiency and OxS worsen during the course of hospitalization, and to establish a temporal scale on recovery (or lack of recovery) of these outcomes over several months post-discharge from the hospital. Nonetheless, this pilot study does provide new information that patients hospitalized for acute ongoing COVID-19 infection have severe GSH deficiency and severely elevated OxS and these could be important targets to consider in the care of such patients. Our study results support the need for future research studies to understand more about redox upheavals in the immediate and delayed aftermath of COVID-19 infection, and trials to understand whether combating GSH deficiency and elevated OxS in acute COVID-19 infection can improve health, prevent complications, and potentially save lives.

## 5. Conclusions

COVID-19 infection is associated with severe intracellular GSH deficiency, elevated oxidative stress and oxidant damage. These defects are present in all age groups including young and middle-aged humans where they are not normally expected. The magnitude of these defects progressively increases with age and is most severe in older humans > 60 year of age. Because GlyNAC supplementation has been shown to be highly effective in correcting GSH deficiency, lowering OxS and oxidant damage in diverse populations including older humans, HIV patients and diabetic patients, it could also improve these defects in patients with COVID-19, and needs to be evaluated in future trials.

## Figures and Tables

**Table 1:** Plasma biochemistry. Values are means ± SD. Means are significantly different at *p* < 0.05.

Parameters	Uninfected Controls	Hospitalized COVID-19 Patients
Hemoglobin (g/L)	13.9 ± 0.8 n = 24	14.0 ± 4.3 *p* = 0.9 n = 59
Total protein (g/dL)	7.2 ± 0.4 n = 24	6.6 ± 0.5 *p* < 0.0001 n = 55
Total bilirubin (mg/dL)	0.7 ± 0.2 n = 24	0.6 ± 0.3 *p* = 0.08 n = 55
Alanine transaminase (U/L)	22.2 ± 7.3 n = 24	54.2 ± 45.5 *p* = 0.001 n = 55
Aspartate transaminase (U/L)	20.0 ± 8.2 n = 24	51.5 ± 56.3 *p* = 0.008 n = 55
Alkaline phosphatase (U/L)	70.2 ± 26.2 n = 24	78.4 ± 38.3 *p* = 0.3 n = 55
BUN (mmol/L)	13.4 ± 3.6 n = 24	17.2 ± 8.2 *p* = 0.03 n = 58
Creatinine (mg/dL)	0.9 ± 0.2 n = 24	0.7 ± 0.2 *p* = 0.0006 n = 58

**Table 2:** GSH and oxidative stress. Data are reported as means ± SD.

Outcome Measure	Controls N = 24	Hospitalized COVID-19 Patients N = 60
RBC-total GSH (mmol/L.RBC)	1.2 ± 0.5	0.5 ± 0.2 *p* < 0.0001
RBC-reduced GSH (mmol/L.RBC)	1.0 ± 0.6	0.4 ± 0.2 *p* < 0.0001
RBC-GSSG (mmol/L.RBC)	0.2 ± 0.2	0.1 ± 0.0 *p* > 0.99
RBC GSH/GSSG	9.4 ± 10.1	8.0 ± 9.2 *p* = 0.5
Plasma TBARS (μM/L)	9.3 ± 9.9	28.2 ± 10.6 *p* < 0.0001
Plasma F2-isoprostane (pg/mL)	93.7 ± 71.0	201.6 ± 51.0 *p* < 0.0001

**Table 3:** Effect of age on intracellular glutathione and plasma biomarkers of oxidative stress and oxidant damage. Data are reported as multiple regression estimates (95% confidence intervals) adjusting for cohort, age group, and sex. *p*-values adjusted for multiple hypothesis tests using Bonferroni correction within each outcome measure. Means are significantly different at *p* < 0.05. YA = young adults (21–40 years), YA-C = young adults with COVID-19 (21–40 years); MA = middle-aged adults (41–60 years), MA-C = middle-aged adults with COVID-19 (41–60 years); OA = older adults (≥60 years), OA-C = older adults with COVID-19 (≥60 years).

Physical Function	Young Adults (21–40 years)	Young COVID-19 Patients (21–40 years) *YA* vs. *YA-C* *YA-C* vs. *OA-C*	Middle-Aged Adults (41–60 years)	Middle-Aged COVID-19 Patients (41–60 years) *MA* vs. *MA-C* *MA-C* vs. *YA-C*	Older Adults (≥60 years)	Older COVID-19 Patients (≥60 years) *OA* vs. *OA-C* *OA-C* vs. *MA-C*
RBC-total GSH (mmol/L.RBC)	1.8 (1.7, 1.9)	0.7 (0.7, 0.8) *p* < 0.0001 *p* < 0.0001	1.1 (1.0, 1.3)	0.3 (0.3, 0.4) *p* < 0.0001 *p* < 0.0001	0.8 (0.6, 0.9)	0.3 (0.2, 0.4) *p* < 0.0001 *p* > 0.99
RBC-reduced GSH (mmol/L.RBC)	1.7 (1.6, 1.8)	0.7 (0.6, 0.7) *p* < 0.0001 *p* < 0.0001	1.0 (0.9, 1.1)	0.3 (0.2, 0.3) *p* < 0.0001 *p* < 0.0001	0.4 (0.3, 0.5)	0.2 (0.1, 0.3) *p* = 0.036 *p* > 0.99
RBC-GSSG (mmol/L.RBC)	0.2 (0.1, 0.2)	0.1 (0.0, 0.1) *p* = 0.09 *p* > 0.99	0.1 (0.0, 0.2)	0.1 (0.0, 0.1) *p* > 0.99 *p* > 0.99	0.4 (0.3, 0.5)	0.1 (0.0, 0.1) *p* < 0.0001 *p* > 0.99
RBC GSH/GSSG ratio	10.0 (4.9, 15.0)	14.7 (11.5, 17.8) *p* = 0.8 *p* < 0.0001	16.7 (11.0, 22.3)	3.7 (0.6, 6.8) *p* = 0.001 *p* < 0.0001	1.9 (0, 7.0)	2.5 (0, 5.7) *p* > 0.99 *p* > 0.99
Plasma TBARS (μM/L)	2.4 (0, 5.9)	18.1 (15.9, 20.3) *p* < 0.0001 *p* < 0.0001	2.7 (0, 6.6)	29.9 (27.8, 32.1) *p* < 0.0001 *p* < 0.0001	23.2 (19.7, 26.7)	39.6 (37.3, 41.8) *p* < 0.0001 *p* < 0.0001
Plasma F2-isoprostane (pg/mL)	45.4 (23.5, 67.4)	162.3 (148.5, 176.0) *p* < 0.0001 *p* < 0.0001	49.7 (25.2, 74.3)	195.9 (182.4, 209.4) *p* < 0.0001 *p* = 0.006	190.1 (168.2, 212.1)	259.8 (245.6, 274.1) *p* < 0.0001 *p* < 0.0001

## Data Availability

All of the data are contained within the article.
